# Rapid point-of-care detection of SARS-CoV-2 infection in exhaled breath using ion mobility spectrometry: a pilot study

**DOI:** 10.1186/s40001-023-01284-3

**Published:** 2023-09-02

**Authors:** Florian Voit, J. Erber, M. Feuerherd, H. Fries, N. Bitterlich, E. Diehl-Wiesenecker, S. Gladis, J. Lieb, U. Protzer, J. Schneider, F. Geisler, R. Somasundaram, R. M. Schmid, W. Bauer, C. D. Spinner

**Affiliations:** 1 Department of Internal Medicine II, University Hospital Rechts Der Isar, School of Medicine, Technical University of Munich, Ismaninger Str. 22, 81675 Munich, Germany; 2 Institute of Virology, Helmholtz Center Munich, TUM, School of Medicine, Munich, Germany; 3 Division of Gastroenterology, Massachusetts General Hospital and Harvard Medical School, Boston, USA; 4 B. Braun Melsungen AG, Melsungen, Germany; 5 ABX-CRO Advanced Pharmaceutical Services Forschungsgesellschaft mbH, Dresden, Germany; 6 Department of Emergency Medicine, Charité-Universitätsmedizin Berlin, Freie Universität Berlin and Humboldt-Universität Zu Berlin, Berlin, Germany; 7 German Center for Infection Research (DZIF), Munich Partner Site, Munich, Germany

**Keywords:** COVID-19, SARS-CoV-2, Breath gas analysis, Ion mobility spectrometry

## Abstract

**Background:**

An effective testing strategy is essential for pandemic control of the novel Coronavirus disease 2019 (COVID-19) caused by infection with severe acute respiratory syndrome coronavirus 2 (SARS-CoV-2). Breath gas analysis can expand the available toolbox for diagnostic tests by using a rapid, cost-beneficial, high-throughput point-of-care test. We conducted a bi-center clinical pilot study in Germany to evaluate breath gas analysis using multi-capillary column ion mobility spectrometry (MCC-IMS) to detect SARS-CoV-2 infection.

**Methods:**

Between September 23, 2020, and June 11, 2021, breath gas measurements were performed on 380 patients (SARS-CoV-2 real-time polymerase chain reaction (PCR) positive: 186; PCR negative: 194) presenting to the emergency department (ED) with respiratory symptoms.

**Results:**

Breath gas analysis using MCC-IMS identified 110 peaks; 54 showed statistically significant differences in peak intensity between the SARS-CoV-2 PCR-negative and PCR-positive groups. A decision tree analysis classification resulted in a sensitivity of 83% and specificity of 86%, but limited robustness to dataset changes. Modest values for the sensitivity (74%) and specificity (52%) were obtained using linear discriminant analysis. A systematic search for peaks led to a sensitivity of 77% and specificity of 67%; however, validation by transferability to other data is questionable.

**Conclusions:**

Despite identifying several peaks by MCC-IMS with significant differences in peak intensity between PCR-negative and PCR-positive samples, finding a classification system that allows reliable differentiation between the two groups proved to be difficult. However, with some modifications to the setup, breath gas analysis using MCC-IMS may be a useful diagnostic toolbox for SARS-CoV-2 infection.

*Trial registration*: This study was registered at ClinicalTrials.gov on September 21, 2020 (NCT04556318; Study-ID: HC-N-H-2004).

**Supplementary Information:**

The online version contains supplementary material available at 10.1186/s40001-023-01284-3.

## Background

Almost 3 years after its emergence, coronavirus disease 2019 (COVID-19), caused by severe acute respiratory syndrome coronavirus 2 (SARS-CoV-2), remains a global burden [1]. In addition to developing vaccines and therapeutic options, an effective testing strategy remains an important component of the pandemic response, particularly with new variants constantly emerging [2, 3]. Fast and reliable test results are essential for pandemic control, especially in healthcare facilities. Particularly in the Emergency Department (ED) setting, screening strategies are key for intra-hospital patient flow, isolation precautions, and avoiding nosocomial outbreaks [4, 5]. To follow antimicrobial stewardship (AMS) concepts, rapidly identifying a viral pathogen as the source of an acute infection is paramount to avoiding antibiotic overuse [6, 7].

Real-time polymerase chain reaction (RT-PCR) from nasopharyngeal swabs is the gold standard for SARS-CoV-2 diagnosis [8, 9]. However, laboratory-based RT-PCR with a turnaround time (TAT) of more than four hours is not applicable for rapid decision-making in the ED, and upcoming point-of-care RT-PCRs with TATs of approximately 20 min minimum are too costly and limited in their availability [10]. SARS-CoV-2 antigen testing can be performed at the point of care and usually provides results after approximately 15 min. However, it should be noted that the sensitivity of antigen testing is significantly lower compared to RT-PCR, especially at low viral loads [11–13]. Moreover, new SARS-CoV-2 variants can escape detection by commonly used antigen tests [14, 15]. Microfluidics-based testing represents a promising future technology, but application at the point of care is still in the early stages of development [16, 17]. Thus, there is a great need for rapid and resource-saving diagnostic alternatives that can be performed at point of care.

In SARS-CoV-2 infection, the upper airways are the main sites of viral replication and inflammation. Therefore, it seems reasonable that the analysis of exhaled breath could represent an attractive, non-invasive approach [18]. Breath gas analysis can quickly and reliably detect volatile organic compounds (VOCs) at their lowest concentrations. The most common methods are ion mobility spectrometry (IMS) and mass spectrometry (MS) [19–22]. In recent years, breath gas analysis has been used to detect various respiratory pathogens [23–27].

The first attempts have already been made to identify a SARS-CoV-2-specific fingerprint in the VOC profile of breath [18]. In a preclinical study, we demonstrated that IMS can discriminate SARS-CoV-2 from other respiratory viruses by analyzing air samples collected from the headspace of virus-infected in vitro cultures [28]. The first small clinical studies on the detection of SARS-CoV-2 in breath gas showed promising results [29–36]. For instance, Ibrahim et al. identified seven exhaled breath features (benzaldehyde, 1-propanol, 3,6-methylundecane, camphene, beta-cubebene, iodobenzene, and an unidentified compound) that distinguished between PCR-positive and PCR-negative patients using regression analysis [32]. In another study by Grassin-Delyle et al. [33] the compounds methylpent-2-enal, 2,4-octadiene, 1-chloroheptane, and nonanal were found to be useful in identifying patients with COVID-19-associated acute respiratory distress syndrome (ARDS).

However, because of the high inter-individual diversity and variability of VOC profiles, studies with a large number of patients are needed to develop a reliable diagnostic test.

In this bi-center pilot study, we investigated whether breath gas analysis using multi-capillary column ion mobility spectrometry (MCC-IMS) could distinguish between symptomatic SARS-CoV-2 PCR-positive (PCR-positive) and -negative (PCR-negative) individuals. For this purpose, we performed breath gas measurements in individuals who presented to the ED with suspected COVID-19. RT-PCR was used as the gold standard to test for SARS-CoV-2, and breath gas measurements of the upper and lower respiratory tracts were performed in 380 participants (186 PCR positive and 194 PCR negative).

## Material and methods

### Materials

**Table:** 

Material	Supplier
SpiroScout PC	Ganshorn, Niederlauer, Germany
PFT-Filter	Ganshorn, Niederlauer, Germany
ScoutTube	Ganshorn, Niederlauer, Germany
Adapter for PFT-Filter	Stromboli, Bochum, Germany
Connecting sample loop	KonMed, Rotkreuz, Switzerland
Incidin Plus 0.5%	Ecolab Deutschland GmbH, Monheim am Rhein, Germany

### Software

VOCan software, B. Braun Melsungen AG, Center of Competence Breath Analysis, Dortmund, Germany.

VisualNow, Version 3.9.2 B. Braun Melsungen AG, Center of Competence Breath Analysis, Dortmund, Germany.

RapidMiner Version 9.2.001, Rapid, Boston, MA, USA.

SPSS V27, IBM, Armonk, NY, USA.

GraphPad Prism 7.04, GraphPad, La Jolla, CA, USA.

### Study design and data acquisition

This case–control accuracy study was conducted at the University Hospital rechts der Isar, Technical University of Munich, Munich, Germany (TUM), and Charité-Universitätsmedizin Berlin, Campus Benjamin Franklin, Germany (CBF). A total of 396 adult (≥ 18 years) participants were recruited from patients presenting to the ED between September 23, 2020, and June 11, 2021 (TUM: 313, CBF: 83). No formal power calculation was performed at the outset of the study for the signals, and the effect sizes were yet to be determined.

The inclusion criteria were signs or symptoms of any respiratory system infection, fever, or radiological findings suggesting pulmonary manifestations of viral lung infection, and performance of reverse transcription-polymerase chain reaction (RT-PCR) testing for SARS-CoV-2. Equal proportions of patients with positive and negative test results were recruited for this study. Major exclusion criteria were inability to perform breath gas measurement, a history of SARS-CoV-2 infection, or participation in another clinical study prior to breath analysis, which could influence the results of the breath analysis according to the assessment of the principal investigator. Data obtained during routine clinical use were documented using an electronic Case Report Form (eCRF). Additionally, predefined clinical signs and symptoms (fever, shivering, cough, dyspnea, headache, limb pain, diarrhea, loss of smell or taste, and fatigue) and demographic and background variables (such as age, sex, and smoking habits) were documented.

Breath gas analysis was performed within 48 h of the RT-PCR testing. 120 min before the breath gas analysis, participants had to refrain from food, liquid intake (except water), or smoking. Written informed consent was obtained from each participant before inclusion in this study.

This study was approved by the Ethics Committee of the Technical University of Munich, School of Medicine, University Hospital rechts der Isar, Munich, Germany (approval no. 437/20 S-KH) and conducted in accordance with the Declaration of Helsinki. This study was registered at ClinicalTrials.gov (NCT04556318; Study-ID: HC-N-H-2004).

### SARS-CoV-2 PCR

SARS-CoV-2 polymerase chain reaction (PCR) was performed using nasopharyngeal swabs. For the Munich site, PCR was performed by detection of the SARS-CoV-2 N gene using established routine diagnostic platforms at the Institute of Virology (Technical University of Munich), such as Cepheid GeneXpert (Sunnyvale, USA), Pathofinder RealAccurate (Maastricht, The Netherlands), or the QIAGEN NeuMoDx (Hilden, Germany) system, and Cycle threshold (Ct) values were available for every patient. For the Berlin site, the SARS-CoV-2 test on the Cobas® 6800 or 8800 system, Cepheid Xpress CoV-2 test, and Cobas® Liat® SARS-CoV-2 test were used.

### Breath gas analysis by MCC-IMS

Breath gas samples for analysis using the MCC-IMS measurement tool were collected using an ultrasound-based spirometer (SpiroScout®, Ganshorn), which consisted of an ultrasound sensor with a disposable breathing tube used for breath sampling. The instrument was connected to a laptop computer with a dedicated analysis program for breath gas analysis (VOCan, B. Braun Melsungen AG, Center of Competence Breath Analysis, Dortmund, Germany). IMS allows the detection of gaseous volatile organic compounds in the trace range of ng/L to pg/L. As a result, gas samples with complex compositions and high moisture contents can be analyzed. The MCC-IMS system (Breath Discovery; B. Braun Melsungen AG, Center of Competence Breath Analysis) combines this highly sensitive method with gas chromatographic pre-separation using a multi-capillary column (1000 capillaries in parallel, inner diameter 40 µm, film thickness 200 nm, type OV-5; Multichrom Ltd., Moscow/Novosibirsk, Russia). A 95 MBq 63Ni β-radiation source was used to ionize the carrier gas (purified room air provided by REDMON, B. Braun Melsungen AG, Germany), which in turn ionized the sample via ion–molecule reactions. The ionized analytes were detected on a Faraday plate at the end of the drift tube by measuring the voltage. The data were displayed in a three-dimensional IMS chromatogram. Here, the peaks are defined by the drift time (via IMS), retention time (via MCC), and signal intensity, which indicates the relative concentration of the analyte.

Two breath samples were collected for each participant. In the first measurement, the breath of the oropharyngeal space was examined. Using SpiroScout, the air between 10 and 500 mL of each exhalation was passed over the sampling loop. Sampling was completed when the collection time added from the individual breaths was 10 s. Sample analysis was performed for 480 s. In the second measurement, the air exhaled from the lungs was analyzed. For this purpose, the sample was collected from the exhaled air, starting at a flow volume of 500 mL until flow reversal (inspiration). The collection time was 10 s, followed by sample analysis. This was followed by disinfection of the device with Incidin Plus 0.5%, a blank measurement to check the purity of the measuring device, and a room air measurement for comparison with the breath measurement.

### Data analysis and statistics

Data analysis of breath gas measurements was performed independently of the RT-PCR results. Manual peak detection was performed using the VisualNow version 3.9 software (B. Braun Melsungen AG, Germany) [28]. The maximum peak values of the PCR-negative and PCR-positive groups and defined subgroups were compared using the Mann–Whitney *U* test.

Decision trees were generated using SPSS V27 [37]. The maximum values of all peaks considered for the decision trees were analyzed according to their diagnostic quality (area under the receiver operating characteristic curve). For each peak, the thresholds for 100% sensitivity and the threshold for 100% specificity were determined. True-positive (TP) and true-negative (TN) results detectable using these thresholds were counted, with a limit on the number of false positives (FPs) and false negatives (FNs) permitted. The peak with the largest sum of TP and TN results was selected (in the case of several peaks with the same sum, the peak with the lowest number in the given order was selected). The records that could be identified as TP and TN according to the selected peak were marked as “scored” and eliminated from the total database. For the remaining records, the procedure was repeated using a reduced database for all peaks that had not yet been considered. The procedure was terminated when no more than 20 records were identified as having TP or TN. The remaining records were classified as positive, with a setting sensitivity of 100% and a specificity of not less than 90% (in the case of 200 patients with negative PCR results). The algorithm was applied again to generate another decision tree using thresholds for lower sensitivity and specificity. The decision tree with the highest Youden index was selected for further consideration. Decision trees were generated for different subgroups based on site, sex, age, and cycle Ct values.

Principal component analysis (PCA) is an orthogonal linear transformation that transforms the given data into a new coordinate system to create a lower-dimensional subspace such that as little information as possible is lost and redundancy is reduced. PCA was used for dimensionality reduction by projecting maximal peak values onto only the first few principal components using SPSS functionality. The dimension was reduced by all components with eigenvalues > 1 in the transformation matrix, and the sum of their variances was 90% of the total variance. Additionally, linear discriminant analysis was performed by systematically selecting the peaks that provided the highest increase in accuracy in a step-by-step manner.

Statistical analyses were performed using SPSS V27 and GraphPad Prism software version 7.04. Statistical analyses were performed as indicated.

## Results

**Fig. 1:**
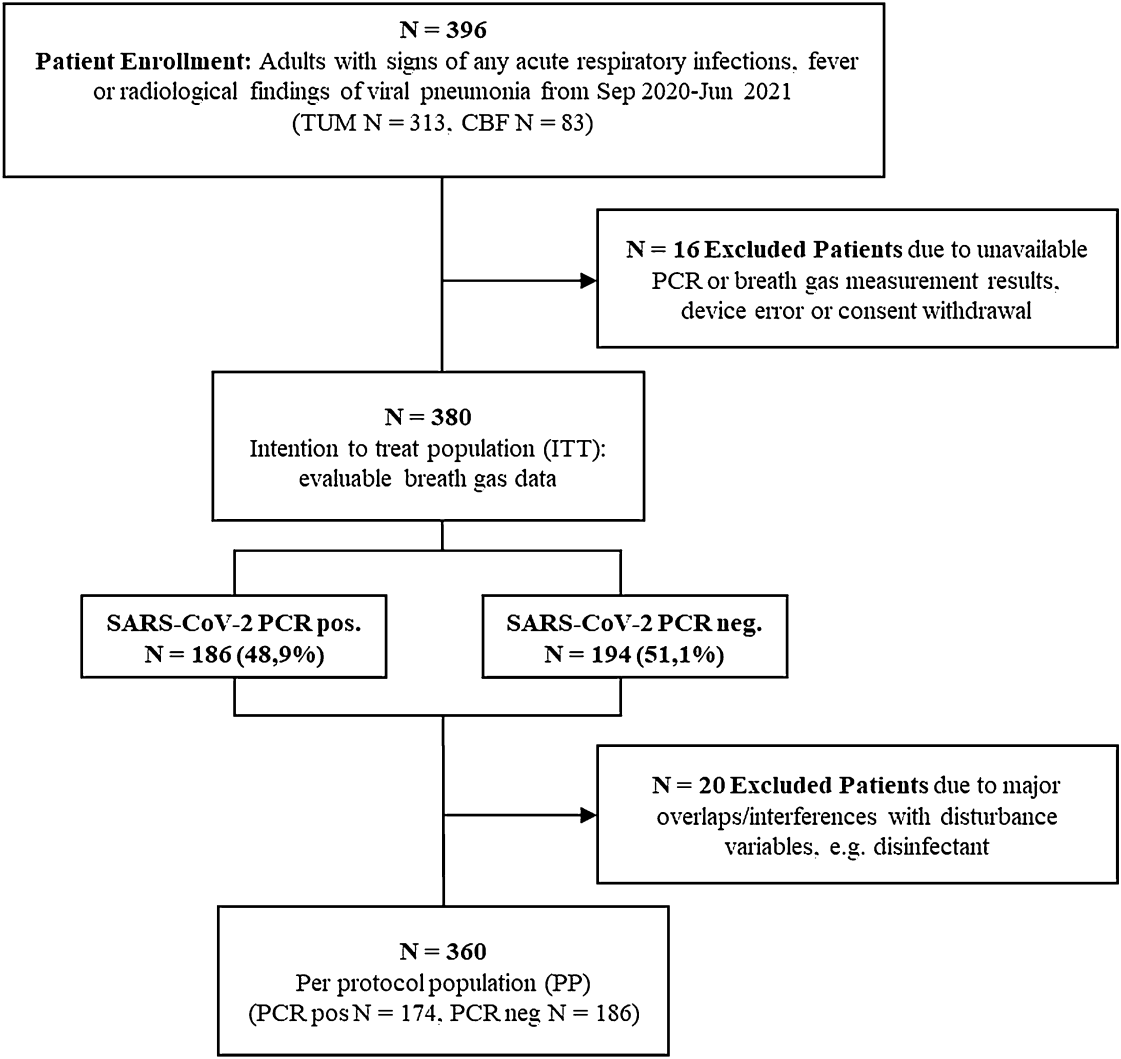
Study profile. Patients were recruited between September 2020 and June 2021 at the Technical University of Munich (TUM) and Charité-Universitätsmedizin Berlin, Campus Benjamin Franklin, Germany (CBF); *N* Number of patients

From September 2020 to June 2021, 396 adult patients with signs of acute respiratory tract infection, fever, or radiological findings suggestive of viral lung infection were recruited from the EDs of TUM and CBF. Three hundred and eighty patients had evaluable breath gas data and PCR results (intention-to-treat population, ITT; Fig. 1). For 360 patients, no major overlaps or interference with disturbance variables was detected (per protocol population, PP).

Demographic and baseline clinical characteristics were relatively balanced between the PCR-positive and PCR-negative groups (Table 1). The mean age was 61.5, with more than half of the patients older than 60 years. The proportion of women was 38.7% (PCR positive) and 36.1% (PCR negative). The proportion of smokers was low overall but significantly higher in the PCR-positive group than in the PCR-negative group (*p* = 0.002, Fisher’s exact test). The median duration of symptoms at inclusion was 4.0 days (interquartile range [IQR], 2.0 to 7.0) in the PCR-positive group and 7.0 (IQR, 4.0 to 8.5) in the PCR-negative group.

**Table 1:** Patient characteristics at baseline

SARS-CoV-2 PCR (sample size, No)	Positive (186)	Negative (194)	Total (380)
Participant no. at study site			
Munich	154	155	309
Berlin	32	39	71
Age—years	58.1 ± 16.2	64.8 ± 17.2	61.5 ± 17.0
Age group ≥ 60—No. (%)	91 (48.9)	127 (65.5)	218 (57.4)
Female sex—No. (%)	72 (38.7)	70 (36.1)	142 (37.3)
Smokers—No. (%)	26 (13.4)	9 (4.8)	35 (9.2)
Median duration of symptoms^a^ (IQR)—days	4.0 (2.0–7.0)	7.0 (4.0–8.5)	5.0 (3.0–8.0)

Plus–minus values are mean ± standard deviation

*No* number of participants, *IQR* interquartile range

^a^Only patients with at least one symptom (*n* = 363)

**Fig. 2:**
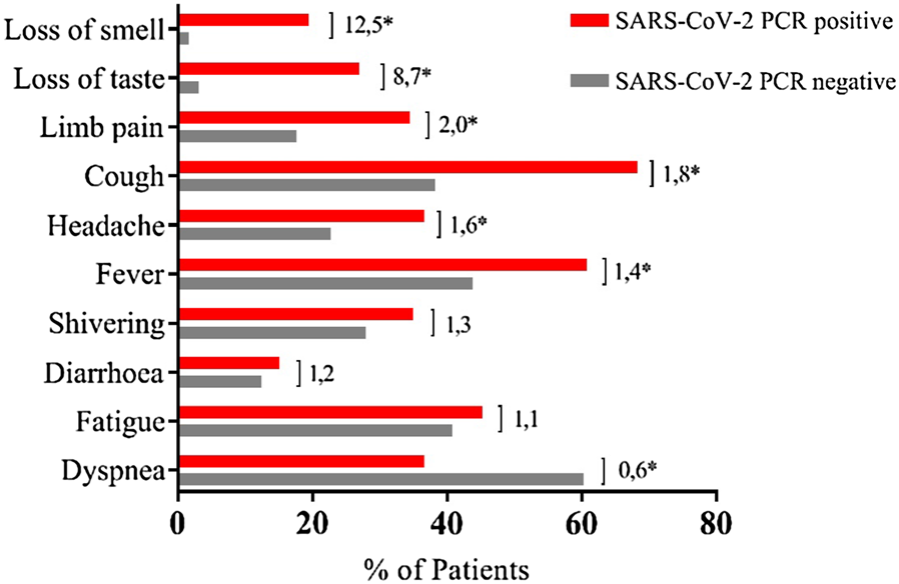
Symptoms. Symptoms are listed in descending order of differential frequency in PCR-positive and -negative patients. It is noted that patients could report more than one symptom. Numbers indicate fold difference between SARS-CoV-2 PCR positive and negative. * significant (*p* < 0.05). Significance was assessed by Fisher’s exact test

Cough and fever were the most common symptoms in the PCR-positive group; however, these complaints were also very common in the PCR-negative group (Fig. 2). However, loss of smell or taste was almost exclusively reported in the PCR-positive group (fold difference 12.5 and 8.7, respectively).

Peripheral oxygen saturation on room air (SpO_2_) and laboratory values of C-reactive protein (CRP), lactate dehydrogenase (LDH), D-dimer, and absolute lymphocyte count were not significantly different between the SARS-CoV-2 PCR-positive and -negative groups (Table 2). Only procalcitonin (PCT), a marker of bacterial infection, was significantly higher in the SARS-CoV-2 PCR-negative group.

**Table 2:** Laboratory tests

	Unit	SARS-CoV-2 PCR negative			SARS-CoV-2 PCR positive			*p*_U_ value
		*N*	Mean (SD)	Median (*Q*1…*Q*3)	*N*	Mean (SD)	Median (*Q*1…*Q*3)	
Procalcitonin	ng/mL	50	2.99 (6.45)	0.40 (0.18 … 2.42)	47	0.78 (3.62)	0.10 (0.10 … 0.20)	< 0.001
C-reactive protein	mg/dL	192	6.64 (7.93)	3.10 (0.62…10.65)	185	5.48 (6.25)	3.10 (1.10…8.10)	0.777
Lactate dehydrogenase	U/L	163	304.1 (151.5)	272.0 (218.0…341.0)	163	310.1 (109.0)	284.0 (237.0…365.0)	0.121
D-dimer	mg/L	85	4.03 (8.10)	1.06 (0.43…3.01)	155	1.35 (2.86)	0.69 (0.42 … 1.19)	0.002
Absolute lymphocyte count	G/L	107	1.13 (1.13)	0.97 (0.52 … 1.34)	172	4.27 (42.63)	0.94 (0.66 … 1.24)	0.622
SpO_2_	%	181	95.4 (14.2)	96.0 (94.0 … 98.0)	172	95.0 (3.7)	95.0 (94.0 … 98.0)	0.167

Laboratory tests for PCR-negative and PCR-positive groups. Statistical significance was assessed by Mann–Whitney *U* test

SpO_2_, peripheral oxygen saturation; SD, standard deviation; *Q*1, first quartile; *Q*3, third quartile; *p*_U_ Mann–Whitney *U* test

Ct values, which are semi-quantitative values of the concentration of viral genetic material in a patient’s sample as determined by RT-PCR, were available for the positive samples at the TUM site [38]. Almost two-thirds of the participants had a Ct value < 30, which is generally considered an infectious viral load (Additional file 1: Fig. S1) [39].

### Breath gas analysis

#### Peak intensity

Breath gas analysis using the IMS measurement tool was performed within 48 h of SARS-CoV-2 RT-PCR testing. For all patients, samples were collected from the oropharyngeal space (throat, T) and lungs (L) (for details, see “Breath gas analysis by MCC-IMS” section).

In the IMS chromatograms, 110 peaks were identified by manual peak selection. Overall, 54 peaks (11 peaks for ITT-L only, 8 for ITT-T only, and 35 for both ITT-L and ITT-T) showed statistically significant differences in peak intensity between the SARS-CoV-2 PCR-negative and -positive groups in the ITT population (Additional file 1: Table S1). Some maxima of peaks showed significant differences in peak intensity between SARS-CoV-2 PCR positive and PCR negative for both throat and lung samples, with similar peak intensities for both study sites (Table 3, Peak 1). For other peaks, differences between sites were observed, although consistent and significant differences between SARS-CoV-2 PCR-positive and PCR-negative samples were still evident (Table 3, Peak 2).

**Table 3:** Exemplary characterization of two maxima of peaks in both lung and throat samples

	Site	SARS-CoV-2 PCR negative		SARS-CoV-2 PCR positive		A_ROC_ (CI)	*p*_U_ (result)
		Mean (SD)	Median (*Q*1 … *Q*3)	Mean (SD)	Median (*Q*1 … *Q*3)		
Peak 1-lung	TUM	17.57 (11.46)	14.72 (10.11 … 21.31)	13.21 (8.73)	11.59 (8.13 … 15.20)	0.357 (0.296 … 0.418)	< 0.001
	CBF	17.32 (8.08)	16.14 (11.48 … 21.80)	12.56 (4.94)	10.65 (8.43 … 16.98)	0.306 (0.183 … 0.428)	0.005
	p_U_ (site)	0.521		0.728			
Peak 1-throat	TUM	18.04 (11.32)	14.55 (11.12 … 21.07)	14.08 (9.88)	11.42 (8.55 … 15.07)	0.346 (0.285 … 0.407)	< 0.001
	CBF	19.10 (10.96)	15.97 (11.74 … 21.65)	13.66 (6.51)	13.50 (8.70 … 15.22)	0.323 (0.198 … 0.447)	0.010
	p_U_ (site)	0.371		0.365			
Peak 2-lung	TUM	9.75 (9.41)	8.70 (6.30 … 11.01)	8.66 (9.25)	7.14 (5.48 … 9.24)	0.374 (0.312 … 0.436)	< 0.001
	CBF	6.85 (3.83)	5.83 (4.75 … 7.62)	5.31 (2.10)	4.67 (4.02 … 6.56)	0.335 (0.207 … 0.462)	0.017
	p_U_ (site)	< 0.001		< 0.001			
Peak 2-throat	TUM	16.23 (19.42)	12.82 (8.97 … 19.09)	12.15 (9.43)	9.88 (7.12 … 14.92)	0.381 (0.318 … 0.443)	< 0.001
	CBF	7.40 (3.77)	6.29 (5.09 … 8.29)	5.85 (1.69)	5.76 (4.47 … 6.84)	0.379 (0.248 … 0.509)	0.080
	p_U_ (site)	< 0.001		< 0.001			

The bar graphs on the left show the distribution of peak intensities in quartiles (red, dark blue: *Q*2; yellow, light blue: *Q*3). The x-axis signal intensity is expressed in arbitrary units ($\times {10}^{3}$). The table on the right shows the statistical characteristics of the peaks. Statistical significance was assessed using the Mann–Whitney *U* test

TUM, Technical University of Munich; CBF, Charité-Universitätsmedizin Berlin, Campus Benjamin Franklin; SD, standard deviation; *Q*1–4, quartile 1–4; CI, confidence interval

#### Decision trees

A commonly used tool in operations research is decision trees, which were also applied in our previous preclinical project (for details, see “Data analysis and statistics” section). For the PP and ITT groups, the sensitivity and specificity were well over 80% for the lung, throat, and combined samples (Table 4).

**Table 4:** Differentiation of breath gas samples by decision tree analysis

Area	*N*	Sensitivity (%)	Specificity (%)
Lung (PP-L)	361	82.9	85.5
Throat (PP-R)	370	85.4	84.9
Lung and Throat (PP)^a^	360	82.8	85.5
Lung (ITT-L)	380	84.4	80.9
Throat (ITT-T)	380	83.9	82.5
Lung and Throat (ITT)^a^	380	85.5	83.0

Decision trees were calculated as described in “Materials and methods” section (“Data Analysis and statistics”)

*PP* per protocol, *ITT* intention-to-treat

^a^Combination by arithmetic mean

We wondered whether the sensitivity of detecting SARS-CoV-2 infection depended on the participant’s viral load. However, there was no trend toward better sensitivity in the group with a Ct value of less than 30 (higher viral load) compared to the groups with a Ct value of 30–35 and a Ct value greater than 35 (Additional file 1: Table S2). However, subgroup analyses implied an influence of site, sex, and age. Combining these influencing variables would reduce the available number of cases to such an extent that decision trees would no longer be meaningful. In addition, despite the relatively high values for sensitivity and specificity, decision trees did not prove robust to changes in the database (e.g., the use of only a part of the dataset).

#### Linear discriminant and principal component analyses

Next, we evaluated the potential of model building using linear discriminant analysis. We selected 11 peaks with the smallest Area under the ROC Curve (AUC_(ROC)_) to differentiate between SARS-CoV-2 PCR-negative and PCR-positive samples from the ITT-L group. For each of these peaks, the comparison between the maximum peak of the SARS-CoV-2 PCR-positive and PCR-negative groups was statistically significant (*p* < 0.001). Linear discriminant analysis classified the two groups with a sensitivity of 74% and specificity of 52% (Table 5).

**Table 5:** Results of linear discriminant analyses and systematic searches for the ITT-L group

Site	Method	Parameters	Sensitivity (%)	Specificity (%)	Accuracy (%)	Youden index (%)
TUM	linear discriminant analyses	10 Peaks	74.7	55.5	65.0	30.2
TUM	systematic search	16 factors	77.9	67.7	72.8	45.6
CBF	linear discriminant analyses	8 Peaks	68.8	53.8	60.6	22.6
CBF	systematic search	14 factors	87.5	79.5	83.1	67.0
Total	linear discriminant analyses	11 Peaks	73.7	51.5	62.4	25.2
Total	systematic search	16 factors	77.4	67.0	72.1	44.4

Linear discriminant analyses and systematic searches were performed as described in “Data Analysis and statistics” section. Youden Index = sensitivity + specificity − 1

Dimensional reduction was attempted using PCA (“Data Analysis and statistics” section). Only three of the 11 peaks mentioned previously had an eigenvector with an eigenvalue greater than 1. With the help of these three factors, a cumulative variance of 78% can be explained. However, the dimension reduction (from 11 to 3) led to a worse classification accuracy than that of the discriminant analysis with the 11 original peak maxima (Additional file 1: Table S3). An extension of the most selective combinations by the single peaks with the highest increase in accuracy was found only by a systematic search, resulting in a sensitivity of 77% and a specificity of 67% (Table 5). Similar results were obtained when only TUM site data were examined. The systematic search for CBF data only revealed a significantly better classification, again stressing the influence of the site (Table 5).

## Discussion

Respiratory tract infections can cause metabolic changes that lead to alterations in the respiratory VOC profile, which enables their potential use in non-invasive breath gas diagnostics. In a preclinical study, we have previously shown the reliable differentiation of SARS-CoV-2 from other respiratory viruses using MCC-IMS analysis of air collected from virus-infected cell cultures [28]. The first clinical studies on breath analysis using gas chromatography–mass spectrometry revealed high sensitivity for detecting SARS-CoV-2 but used only small sample sizes (reviewed in [40]). A Dutch study of 4510 non-hospitalized participants also found a high sensitivity for SARS-CoV-2 detection by breath gas analysis with eNose, which uses pattern recognition in cross-reactive metal oxide semiconductor sensors [36]. Using similar technology, Nurputra et al. reported high accuracy in detecting SARS-CoV-2 in samples taken from 43 COVID-19-positive and 40 -negative patients [41].

In our study, 380 participants underwent breath gas analysis using MCC-IMS in both lung and throat spaces. The inclusion criteria were defined as comparable to the established test routine for suspected SARS-CoV-2 infection: symptoms of any respiratory system infection, fever, or radiological findings for viral pneumonia to achieve high consistency with real-world clinical practice.

Demographic and baseline clinical characteristics were overall relatively similar between the PCR-positive and PCR-negative groups. The proportion of smokers was significantly higher in the PCR-positive group. However, considering the overall low smoking rate, the selection bias can be considered to be low. Also, the duration of symptoms upon inclusion was significantly shorter in the PCR-positive group when compared to the PCR-negative group. As this study was conducted as a pilot, measurements were limited to a single time point. The duration of symptoms at the time of inclusion varied widely and showed considerable overlap between the two groups, with interquartile ranges (IQR) of 2–7 days for the PCR-positive group and 4–8.5 days for the PCR-negative group. The VOC profile probably undergoes certain changes during the course of the infection. To gain a more precise understanding of these changes, longitudinal measurements throughout the entire duration of the infection would be desirable. However, for clinical utility, it is crucial that a specific VOC profile remains detectable over an extended period throughout the infection.

Furthermore, consistent with the literature, loss of smell or taste was almost exclusively reported in the PCR-positive group [42]. There were no significant differences in laboratory values between the two groups, except for PCT, which was higher in the PCR-negative group, as expected, due to the probably higher incidence of bacterial infections.

Breath gas analysis using MCC-IMS identified 54 peaks with a statistically significant difference in peak intensity between the PCR-negative and PCR-positive groups. However, finding a classification system that allows reliable differentiation between the two groups is challenging. An algorithm based on decision trees allowed the detection of SARS-CoV-2 with a sensitivity and specificity well above 80%. Subgroup analyses implied the influence of the study site, sex, and age. However, when the generation of decision trees was restricted to a selection of the available datasets, the transferability to the remaining datasets was limited. Furthermore, an influence of the viral load on sensitivity could not be shown. A major cause could be that, despite statistically significant differences in the peak values of SARS-CoV-2 PCR negative and PCR positive, only a few values can be correctly assigned based on a threshold value without simultaneously obtaining incorrect assignments.

Next, we applied a model-building approach using a linear discriminant analysis. In this analysis, only modest values for sensitivity (73.7%) and specificity (51.5%) were obtained. Applying PCA in combination with a systematic search showed a sensitivity of 77.4% and a specificity of 67.0% for SARS-CoV-2-PCR positivity. However, the results depend on the current database and can therefore be assumed not to be transferable to other datasets. Furthermore, it is unclear how a suitable peak can be selected using an algorithm.

As another analysis method, we explored the use of artificial intelligence to create a classification system. However, training computer vision machine learning models (ResNet18, Convolutional Neural Network [43, 44]) on breath gas data failed. Possible reasons for this were the low sample numbers for this initially unplanned analysis and the data points containing little discriminative information from a computer vision perspective.

A sufficient sample size is always a challenge for model building. The inclusion of 380 participants, 186 of whom were PCR positive, in an emergency room setting is remarkable, but especially for a machine learning approach, much higher patient numbers would have been necessary. Highly sensitive breath gas analysis is susceptible to environmental factors such as indoor air or disinfectant used, which became evident in our data because of the influence of the site on peak intensity. This highlights the need for multiple sites to develop a robust algorithm that is not overfitted for one site, and demonstrates the limited transferability of other monocentric breath gas studies.

During the recruitment period of this study, the ancestral variant initially prevailed, but in the first half of 2021, it was increasingly replaced by the Alpha variant (B.1.1.7.) [45]. The concept of breath gas analysis is based on the idea that metabolic changes create specific VOC profiles depending on the pathogen involved (as described in [26, 27]). It would be plausible to assume that different variants of SARS-CoV-2 may potentially generate slightly different VOC profiles. However, it is also reasonable to speculate that an association with the species SARS-CoV-2 should still be possible. Calibration to the currently predominant variant may be advisable to increase the accuracy of the test.

The present study distinguishes itself from other similar projects due to its significantly larger sample size and the inclusion of two study sites (see [31–33]). Moreover, we regard the apparent selection bias, the recruitment of symptomatic individuals from the emergency department, to be a strength of the study as it represents a highly medically relevant group. These factors enhance the statistical power, generalizability, and clinical significance of the findings, making it a valuable contribution to the field of breath gas analysis for SARS-CoV-2 detection.

## Conclusion

In this bi-center clinical pilot study, we demonstrate that breath gas analysis with MCC-IMS allows the detection of SARS-CoV-2 infection in patients with a sensitivity and specificity of more than 80% in a real-life ED setting. However, the algorithms used in the analysis depend on the current database, which makes the transferability of the results to other datasets difficult. Despite the limited robustness of the algorithms developed in this study, the large number of significantly different peaks between the PCR-positive and PCR-negative groups seems promising. The use of drift tubes adapted to the now-known distribution spectrum of the peaks could lead to a much better separation of the frequently superimposed peaks in this dataset. In addition, structural modifications of the measuring device to improve its disinfect ability could reduce the disturbing influence of disinfectants.

This study showed the general feasibility of such a non-invasive point-of-care diagnostic test without the need for a high number of consumables. SARS-CoV-2 has highlighted the role of rapid diagnostic testing in pandemic control. Further research on technical advancements, analysis of highly complex datasets and evaluation in a multicenter environment are required to make breath gas analysis a valuable tool in the future.

### Supplementary Information

**Additional file 1: Table S1.** Significantly different peaks between the PCR-positive and PCR-negative groups for lung and throat samples of the ITT population. **Table S2.** Sensitivity of SARS-CoV-2 detection in decision tree analysis for different groups of Ct values. **Table S3.** Performance of discriminant analysis for reduction of dimensions via principal component analysis (ITT-L). **Fig. S1.** SARS-CoV-2 PCR was performed as described in “Materials and methods” section. Ct values were available for the Munich site only. A lower Ct value indicates higher concentrations of the detected virus genome.

## Data Availability

Written informed consent was obtained from all participants before inclusion in this study. The datasets generated and analyzed during the current study are available from the co-author Heike Fries (email: heike.fries@bbraun.com) upon reasonable request.

## Abbreviations

AMS: Antimicrobial stewardship
AUC(ROC): Area under the ROC Curve
CBF: Charité: Universitätsmedizin Berlin, Campus Benjamin Franklin, Germany
COVID-19: Coronavirus disease 2019
CRP: C-reactive protein
Ct: Cycle threshold
eCRF: Electronic Case Report Form
ED: Emergency department
FN: False negative
FP: False positive
IMS: Ion mobility spectrometry
IQR: Interquartile range
ITT: Intention-to-treat
L: Lung
LDH: Lactate dehydrogenase
MCC-IMS: Multi-capillary column ion mobility spectrometry
MS: Mass spectrometry
PCA: Principal component analysis
PCR: Polymerase chain reaction
PCT: Procalcitonin
PP: Per protocol
RT-PCR: Real-time polymerase chain reaction
SARS-CoV-2: Severe acute respiratory syndrome coronavirus 2
SD: Standard deviation
SpO2: Peripheral oxygen saturation on room air
T: Throat
TAT: Turnaround time
TN: True negative
TP: True positive
TUM: University Hospital rechts der Isar, Technical University of Munich, Germany
VOC: Volatile organic compound
