# Supplementary material for: Rapid point-of-care detection of SARS-CoV-2 infection in exhaled breath using ion mobility spectrometry: a pilot study
Source: Eur J Med Res. 2023 Sep 2;28:318. doi: 10.1186/s40001-023-01284-3 (PMC10474630; doi:10.1186/s40001-023-01284-3)
Supplement: Supplementary file 1 — Additional file 1: Table S1. Significantly different peaks between the PCR-positive and PCR-negative groups for lung and throat samples of the ITT population. Table S2. Sensitivity of SARS-CoV-2 detection in decision tree analysis for different groups of Ct values. Table S3. Performance of discriminant analysis for reduction of dimensions via principal component analysis (ITT-L). Fig. S1. SARS-CoV-2 PCR was performed as described in “Materials and methods” section. Ct values were available for the Munich site only. A lower Ct value indicates higher concentrations of the detected virus genome. [file 40001_2023_1284_MOESM1_ESM.docx]

# Additional Figures and Tables

Table S1. Significantly different peaks between the PCR-positive and PCR-negative groups for lung and throat samples of the ITT population

| **Peak** | **Lung** | | | | | | **Throat** | | | |
| --- | --- | --- | --- | --- | --- | --- | --- | --- | --- | --- |
|  | TUM | | CBF | | | | TUM | | CBF | |
|  | p_U_ | AUC_ROC_ | p_U_ | | | AUC_ROC_ | p_U_ | AUC_ROC_ | p_U_ | AUC_ROC_ |
| C01 | **0.004** | 0.404 |  | |  | |  |  |  |  |
| C02 |  |  | **0.019** | | 0.663 | |  |  |  |  |
| C13 | **<0.001** | 0.383 |  | |  | | **<0.001** | 0.369 |  |  |
| C15 | **0.010** | 0.415 |  | |  | | **0.012** | 0.417 |  |  |
| C16 | **0.003** | 0.404 |  | |  | | **0.002** | 0.398 |  |  |
| C18 | **0.006** | 0.409 |  | |  | | **0.043** | 0.433 |  |  |
| C19 | **0.007** | 0.412 |  | |  | | **<0.001** | 0.358 |  |  |
| C21 | **0.012** | 0.582 |  | |  | | **0.021** | 0.576 |  |  |
| C22 | **<0.001** | 0.352 |  | |  | | **<0.001** | 0.344 |  |  |
| C34 |  |  | **0.021** | | 0.341 | | **0.035** | 0.431 |  |  |
| C35 | **<0.001** | 0.354 |  | |  | | **<0.001** | 0.349 |  |  |
| C36 |  |  |  | |  | | **0.010** | 0.416 |  |  |
| C37 |  |  |  | |  | | **0.003** | 0.403 |  |  |
| C38 |  |  | **0.011** | | 0.324 | |  |  |  |  |
| C39 | **0.004** | 0.405 |  | |  | | **0.001** | 0.392 |  |  |
| C41 |  |  |  | |  | | **0.014** | 0.419 |  |  |
| C44 | **0.001** | 0.395 |  | |  | | **<0.001** | 0.368 |  |  |
| C47 | **0.007** | 0.411 |  | |  | | **<0.001** | 0.377 |  |  |
| C49 | **0.031** | 0.429 |  | |  | | **0.031** | 0.429 |  |  |
| C50 |  |  |  | |  | | **0.014** | 0.419 |  |  |
| C52 | **0.017** | 0.422 |  | |  | | **0.004** | 0.406 |  |  |
| C53 |  |  |  | |  | | **0.036** | 0.431 |  |  |
| C54 | **0.026** | 0.427 |  | |  | | **0.003** | 0.402 |  |  |
| C55 |  |  |  | |  | | **0.048** | 0.435 |  |  |
| C56 | **0.021** | 0.424 |  | |  | | **0.014** | 0.419 |  |  |
| C57 | **0.001** | 0.394 | **0.031** | | 0.350 | | **0.018** | 0.422 | **0.048** | 0.363 |
| C58 | **0.037** | 0.569 |  | |  | |  |  | **0.043** | 0.359 |
| C60 | **0.001** | 0.386 |  | |  | | **0.005** | 0.407 |  |  |
| C63 | **<0.001** | 0.360 | **0.010** | | 0.321 | | **<0.001** | 0.379 | **0.008** | 0.317 |
| C64 |  |  | **0.016** | | 0.333 | |  |  |  |  |
| C65 | **0.004** | 0.404 |  | |  | | **0.012** | 0.418 | **0.031** | 0.351 |
| C68 |  |  | **0.033** | | 0.353 | |  |  |  |  |
| C69 | **<0.001** | 0.362 | **0.031** | | 0.350 | | **<0.001** | 0.375 |  |  |
| C70 | **<0.001** | 0.366 | **0.007** | | 0.312 | | **<0.001** | 0.366 | **0.015** | 0.332 |
| C71 | **0.004** | 0.404 |  | |  | | **0.014** | 0.419 |  |  |
| C72 | **<0.001** | 0.374 | **0.017** | | 0.335 | | **<0.001** | 0.381 |  |  |
| C73 | **<0.001** | 0.357 | **0.005** | | 0.306 | | **<0.001** | 0.346 | **0.010** | 0.323 |
| C77 |  |  |  | |  | | **0.050** | 0.435 |  |  |
| C79 | **0.045** | 0.566 |  | |  | |  |  |  |  |
| C80 | **<0.001** | 0.348 | **0.013** | | 0.328 | | **<0.001** | 0.353 |  |  |
| C82 |  |  |  | |  | | **0.017** | 0.579 |  |  |
| C86 | **<0.001** | 0.344 |  | |  | | **<0.001** | 0.378 |  |  |
| C87 | **<0.001** | 0.376 | **0.047** | | 0.363 | | **0.001** | 0.388 |  |  |
| C90 | **0.001** | 0.387 |  |  | | | **0.003** | 0.403 |  |  |
| P01 | **0.030** | 0.571 |  |  | | | **0.038** | 0.568 |  |  |
| P02 | **<0.001** | 0.361 |  |  | | | **<0.001** | 0.367 |  |  |
| P03 |  |  |  |  | | | **<0.001** | 0.385 |  |  |
| P04 |  |  |  |  | | | **0.010** | 0.585 |  |  |
| P07 |  |  | **0.049** | 0.363 | | | **0.001** | 0.606 |  |  |
| P14 | **0.012** | 0.417 |  |  | | |  |  |  |  |
| P15 | **0.001** | 0.390 |  |  | | |  |  |  |  |
| P16 |  |  |  |  | | | **0.010** | 0.416 |  |  |
| P18 | **0.004** | 0.405 |  |  | | | **0.003** | 0.403 |  |  |
| P19 |  |  |  |  | | | **0.002** | 0.397 |  |  |

Table S2 Sensitivity of SARS-CoV-2 detection in decision tree analysis for different groups of Ct values

| Area | Ct-value < 30 | 30 ≤ Ct-value ≤ 35 | Ct-value > 35 |
| --- | --- | --- | --- |
| PP-L | 83.5% (N=91) | 82.1% (N=28) | 85.2% (N=27) |
| PP-T | 85.7% (N=91) | 89.3% (N=28) | 88.9% (N=27) |
| ITT-L | 86.5% (N=96) | 85.7% (N=28) | 78.6% (N=28) |
| ITT-T | 87.5% (N=96) | 82.1% (N=28) | 78.6% (N=28) |

Table S3 Performance of discriminant analysis for reduction of dimensions via principal component analysis (ITT-L)

| Parameters | Sensitivity | Specificity | Accuracy | Youden Index |
| --- | --- | --- | --- | --- |
| 11 Peaks | 73.7% | 51.5% | 62.4% | 25.2% |
| Factors 1-3 | 74.2% | 46.9% | 60.3% | 21.1% |
| Factors 1-6 | 74.2% | 46.4% | 60.0% | 20.6% |
| Factors 1-11 | 73.7% | 51.5% | 62.4% | 25.2% |

| **Ct values (Munich only)** | | | | | |
| --- | --- | --- | --- | --- | --- |
| N | Mean | SD | Min | Median | Max |
| 152 | 27.8 | 6.6 | 14 | 28.0 | 42 |

Fig. S1 SARS-CoV-2 PCR was performed as described in the Methods section. Ct values were available for the Munich site only. A lower Ct value indicates higher concentrations of the detected virus genome.
